# Regulation of inflorescence architecture by cytokinins

**DOI:** 10.3389/fpls.2014.00669

**Published:** 2014-11-24

**Authors:** Yingying Han, Haibian Yang, Yuling Jiao

**Affiliations:** ^1^State Key Laboratory of Plant Genomics, National Center for Plant Gene Research, Institute of Genetics and Developmental Biology – Chinese Academy of SciencesBeijing, China; ^2^University of Chinese Academy of SciencesBeijing, China

**Keywords:** branching, cytokinin, floral meristem, inflorescence, shoot apical meristem

## Abstract

In flowering plants, the arrangement of flowers on a stem becomes an inflorescence, and a huge variety of inflorescence architecture occurs in nature. Inflorescence architecture also affects crop yield. In simple inflorescences, flowers form on a main stem; by contrast, in compound inflorescences, flowers form on branched stems and the branching pattern defines the architecture of the inflorescence. In this review, we highlight recent findings on the regulation of inflorescence architecture by cytokinin plant hormones. Results in rice (*Oryza sativa*) and *Arabidopsis thaliana* show that although these two species have distinct inflorescence architectures, cytokinins have a common effect on inflorescence branching. Based on these studies, we discuss how cytokinins regulate distinct types of inflorescence architecture through their effect on meristem activities.

## INTRODUCTION

Plants have an enormous, striking diversity of forms, with varying numbers and arrangements of organs in different sizes and shapes; this diversity derives from regulation of meristem activity. The aerial organs of a plant come from the shoot apical meristem (SAM) which gives rise to leaves, stem, and axillary meristems during the vegetative stage and transforms into the inflorescence meristem (IM) after the floral transition. The various developmental patterns of the IM in different species produce diverse inflorescence architectures, which not only attract artists and plant scientists, but also draw the attention of plant breeders, because inflorescence traits directly affect crop yields. Branching hierarchy and complexity depend on the species, but are also affected by environmental factors, including nutrition, light, and temperature (Tanaka et al., 2013; Kyozuka et al., 2014; Teo et al., 2014).

**FIGURE 1:**
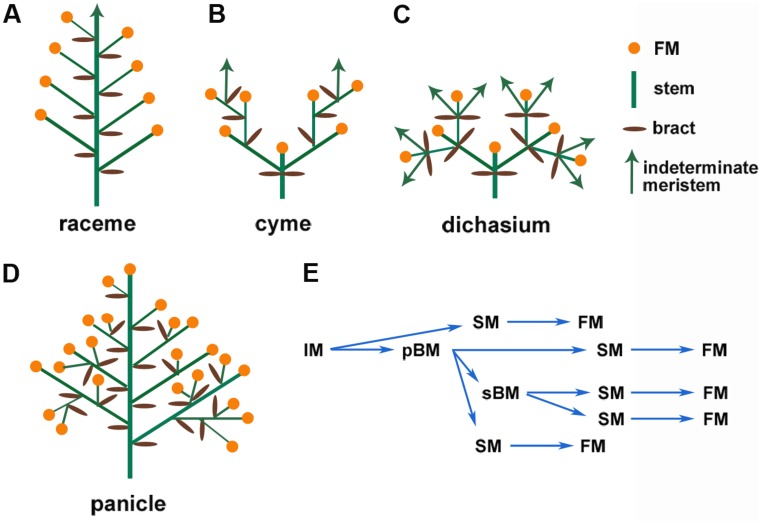
**Schematic representation of common types of inflorescences. (A)** Simple raceme, which is indeterminate and unbranched, **(B)** cyme, **(C)** dichasium, which are determinate and branched, **(D)** panicle, which is determinate and branched, and **(E)** Transition of the reproductive meristems in rice panicle.

The enormous diversity of inflorescence architecture also leads to difficulties in defining consensus criteria to classify these structures. Following Weberling’s (1989) suggestions, inflorescence architectures can be broadly grouped into inflorescences without branching (simple) and inflorescences with branching (compound). Another key parameter is whether the IM ends in a terminal flower (determinate) or continues to produce structures, including branches and flowers (indeterminate). Following these key distinctions, at least three typical groups of inflorescence architectures are commonly seen, namely the raceme (simple, indeterminate, as in *Arabidopsis*), the cyme (complex, determinate, as in tomato), and the panicle [complex, determinate, as in wheat (*Triticum aestivum*); or complex, indeterminate, as in maize (*Zea mays*), especially tassel; **Figure 1**; Prusinkiewicz et al., 2007; Kellogg et al., 2013]. These distinct inflorescence architectures result from different developmental programs that are elaborated below.

Development of the IM conditions the branching of the inflorescence. In *Arabidopsis*, the IM directly initiates floral meristems (FMs, which are determinate meristems) on its flanks; this forms a simple raceme (**Figure 1A**; Benlloch et al., 2007; Tanaka et al., 2013; Teo et al., 2014). The grasses have more diverse inflorescence architectures (Kellogg et al., 2013). In a generalized grass inflorescence, the IM gives rise to several branch meristems (BMs, which are usually indeterminate meristems). These BMs may initiate secondary BMs to form lateral branches and spikelet meristems (SMs) that then initiate FMs (**Figure 1E**). In maize and other Andropogoneae species, determinate spikelet-pair meristems (SPMs) are produced from the IM or BMs, and each SPM makes two SMs. The SM initiates one or more FMs (Kellogg et al., 2013; Kyozuka et al., 2014). These intermediate BMs cause secondary or higher-order branches, which form a compound inflorescence termed the panicle (Benlloch et al., 2007). Therefore, the branch structure determines the final inflorescence pattern, which contributes to the enormous diversity of inflorescence architectures. Specific genetic regulatory networks control every stage and transition of meristem activity, as described in several recent reviews (Tanaka et al., 2013; Kyozuka et al., 2014).

Meristem activity, especially determinacy, fundamentally affects inflorescence architecture. For example, in the raceme-type inflorescence of *Arabidopsis*, the IM continues to initiate FMs; by contrast, in the cyme-type inflorescence of tomato, the IM forms a terminal flower immediately after developing a new IM below it, which reiterates this pattern (). The panicle-type inflorescence is initially indeterminate and initiates BMs and FMs before it finally terminates in a FM in some species. At least two groups of genes, relatives of *Arabidopsis LEAFY* (*LFY*) and *TERMINAL FLOWER1 (TFL1*), play a central role in meristem determinacy. *LFY* promotes determinate FM identity and termination of IMs, and *TFL1* maintains the indeterminacy of IMs to prevent termination (Prusinkiewicz et al., 2007).

Recent work has identified cytokinins as key regulators of inflorescence architecture in plants with different inflorescence types, through regulation of meristem activity, which is often also associated with meristem identity. Cytokinins have profound effects on plant development and growth, including meristem activity (Kyozuka, 2007; Werner and Schmulling, 2009; Perilli et al., 2010). Accumulating data point to a role for cytokinins in influencing inflorescence complexity by fine-tuning IM and BM determinacy. Also, recent work reveals that cytokinins can regulate the initiation of meristems from floral organ axils (the junction where the floral organ meets the stem), and thus convert a determinate flower into an inflorescence (Han et al., 2014). Here we review these two mechanisms through which cytokinins regulate inflorescence architecture.

## CYTOKININS PROMOTE IM ACTIVITY

Increasing cytokinin concentrations and signaling activity increase meristem size and activity. Reduced meristem activity often leads to conversion of an IM or a BM into a terminal flower, which subsequently affects inflorescence architecture.

Work in rice and in *Arabidopsis* showed that cytokinin levels affects meristem activity and inflorescence complexity. The ATP/ADP isopentenyl transferases (IPTs) catalyze the first step of cytokinin biosynthesis (Miyawaki et al., 2006). *Arabidopsis atipt3 5 7* triple mutants and *atipt1 3 5 7* quadruple mutants have lower levels of cytokinins, which leads to reduced IM size, formation of a terminal flower, and conversion of an indeterminate inflorescence to a determinate inflorescence (Miyawaki et al., 2006). Rice *LONELY GUY* (*LOG*) encodes a cytokinin-activating enzyme catalyzing the final step of cytokinin biosynthesis and *LOG* is strongly expressed in BMs and FMs of developing panicles. The absence of *LOG* results in early termination of IM and BMs, which reduces branching complexity (Kurakawa et al., 2007). *Arabidopsis* has nine *LOG* homologs and the triple *log3 log4 log7* and septuple *log1 log2 log3 log4 log5 log7 log8* mutants produce fewer FMs, suggesting reduced IM activity (Kuroha et al., 2009; Tokunaga et al., 2012).

In addition to cytokinin homeostasis, defects in cytokinin signaling also leads to simplified inflorescence architecture. Cytokinins are perceived by transmembrane histidine kinase receptors, such as *Arabidopsis* HISTIDINE KINASE 2 (AHK2), AHK3, and AHK4. The *ahk* triple mutants have a smaller IM that terminates early, resulting in a simplified inflorescence with only a few flowers (Nishimura et al., 2004).

Conversely, elevated cytokinin homeostasis results in increased inflorescence complexity. Cytokinin oxidase/dehydrogenase (CKX) plays a major role in the degradation of bioactive cytokinins (Mok and Mok, 2001). *Arabidopsis* plants overexpressing *CKX1* or *CKX3* have dramatically reduced cytokinins contents and IMs that produce very few flowers (Werner et al., 2003). *CKX* overexpression in tobacco plants also leads to fewer flowers and conversion of IMs from indeterminate to determinate (Werner et al., 2001). Similarly, rice varieties with lower *OsCKX2* expression have more elaborated and larger panicles with more primary and secondary branches and higher yield, and rice varieties with higher *OsCKX2* activity have the opposite phenotype, with fewer branches and lower yield (Ashikari et al., 2005; Li et al., 2013).

Cytokinins promote IM activity and affect inflorescence architecture by promoting expression of the meristematic gene *WUSCHEL* (*WUS*) and suppressing the meristem inhibitors *CLAVATA1* (*CLV1*) and *CLV3*. Plants ectopically treated with cytokinins show a *clv*-like phenotype with larger IMs and more floral organs (Venglat and Sawhney, 1996; Lindsay et al., 2006). Cytokinins suppress the expression of *CLV1*; this suppression results in upregulation of *WUS* expression (Brand et al., 2000; Schoof et al., 2000; Lindsay et al., 2006; Gordon et al., 2009). In addition, cytokinins directly induce *WUS* expression, independent of *CLV1*, and *WUS* enhances cytokinin signaling, forming a positive feedback loop (Leibfried et al., 2005; Gordon et al., 2009). Computational modeling shows that a combination of the negative feedback between *WUS* and *CLV*, and the positive feedback of *WUS* and cytokinin signaling determines the fine-scale positioning of the *WUS*-expressing stem cell niche domain (Gordon et al., 2009; Chickarmane et al., 2012).

## CYTOKININS PROMOTE LATERAL INDETERMINACY IN DETERMINATE FMs

**FIGURE 2:**
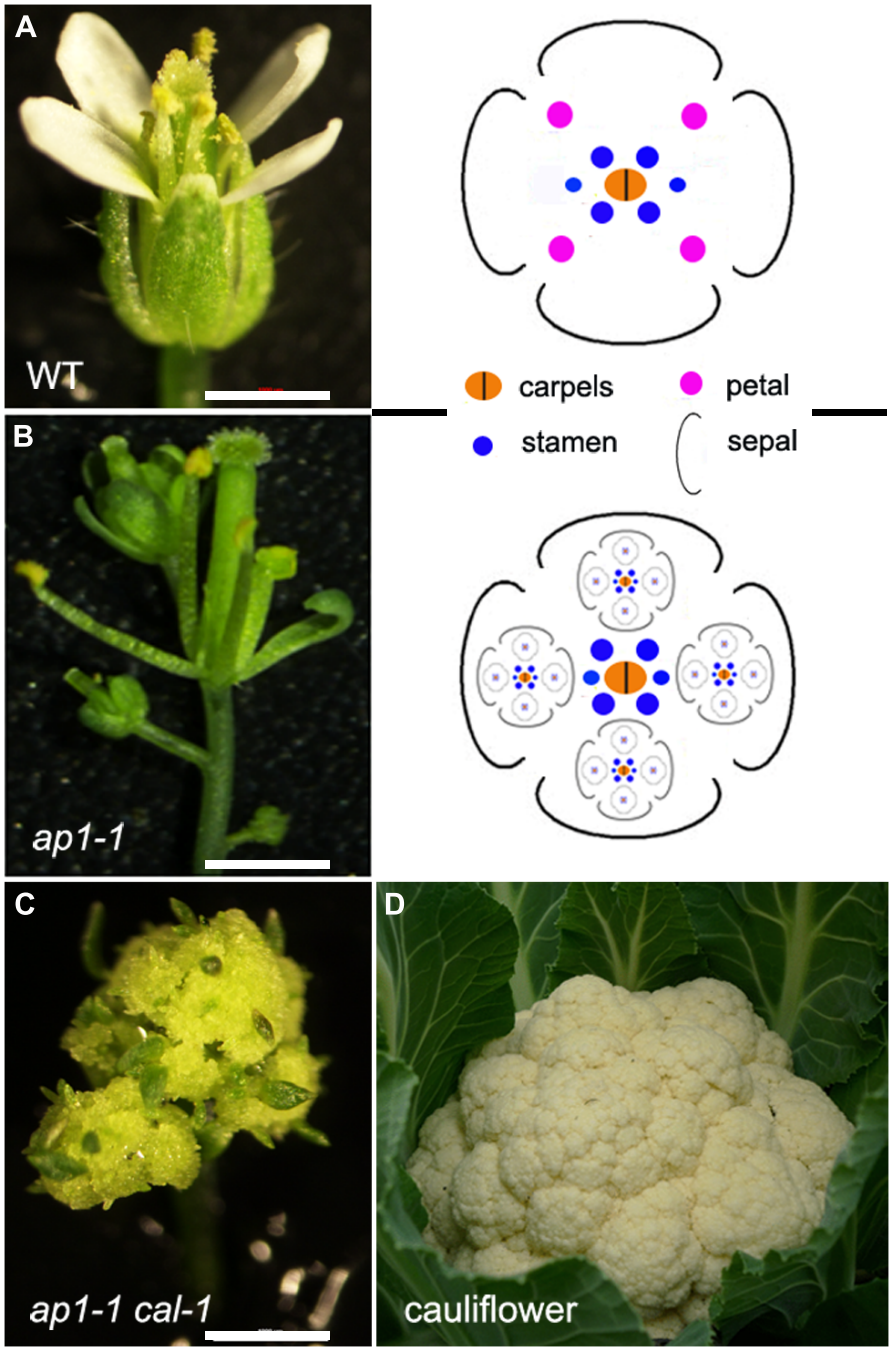
**Conversion of a terminate flower into a pleiochasium-like inflorescence.** Phenotype of *Arabidopsis* **(A)** Lansberg *erecta* wild-type, **(B)** *ap1-1* **(C)** *ap1-1 cal-1* flowers, and **(D)** a cauliflower with a highly elaborated inflorescence, similar to that seen in the *Arabidopsis ap1-1 cal-1* mutant. Bars = 1 mm.

In indeterminate inflorescences, the periphery of the meristem produces BMs (and also SPMs and SMs for grasses) or FMs. In many determinate inflorescences, such as in wheat spikes, BM, SM, and FM can also initiate from the IM before its termination in a FM. In contrast to this initiation pattern, FM and BM can also initiate laterally from a terminal flower, either from the axil of a leaf-like organ (such as petals) or can initiate without subtending lateral organs. These types of inflorescence are termed dichasium and pleiochasium (**Figure 1C**), depending on the number of lateral branches, and can be considered a specialized cyme. Common examples include cauliflower and broccoli, which have a phenotype similar to that of the *Arabidopsis apetala1-1 cauliflower-1* (*ap1-1 cal-1*) double mutants (**Figure 2**). In the *ap1-1* single mutant (**Figure 2B**), secondary flowers laterally initiate from sepal axils and from the pedicel. The *ap1-1 cal-1* double mutants have the same but more complicated inflorescence branching pattern. This lateral inflorescence branching mechanism has many similarities to vegetative stage lateral shoot branching. In contrast to vegetative shoot branching, inflorescences like the raceme and panicle develop iteratively, similar to frond development in ferns (Sanders et al., 2011). Despite these differences, cytokinins also regulate this type of lateral inflorescence branching.

Lateral inflorescence branching is controlled by *AP1* and related MADS-box transcription factor genes in *Arabidopsis* and other Brassicaceae species. In the simple indeterminate inflorescence of *Arabidopsis*, the IM gives rise to FMs and each FM differentiates into four whorls of floral organs that occupy precise positions (**Figure 2A**). In addition to promoting FM formation and outer floral whorl specification of sepals and petals, *AP1* inhibits sepal axil meristem activity (Irish and Sussex, 1990; Mandel et al., 1992). In *ap1* mutants, secondary flowers initiate in the axils of sepals, and tertiary flowers can initiate in the sepal axils of secondary flowers, and so on (**Figure 2B**; Irish and Sussex, 1990; Mandel et al., 1992). This forms a dichasium or pleiochasium-like inflorescence (**Figure 1C**). The inflorescence phenotype in *ap1* is enhanced by *cauliflower* and *fruitful* mutants to form a cauliflower-like, highly elaborated pleiochasium inflorescence (**Figure 2C**; Ferrandiz et al., 2000). Indeed, cauliflower has lost a homolog of *AP1* (**Figure 2D**; Kempin et al., 1995), suggesting that *AP1* function is required to inhibit conversion of a simple raceme to a pleiochasium.

A recent study has shown that *AP1* inhibits lateral inflorescence branching by reducing cytokinin levels. During vegetative stages, leaf axil axillary meristem formation requires cytokinin signaling (Wang et al., 2014) and during reproductive stages, lateral FM formation similarly requires cytokinin signaling (Han et al., 2014). The *ap1* flowers have enhanced cytokinin signaling, as shown by examination of cytokinin-responsive reporter genes, and these flowers also have elevated levels of certain types of cytokinins. In addition, cytokinin treatment or ectopic expression of the cytokinin biosynthesis enzyme *IPT8* in the *AP1*-expressing domain phenocopies the sepal axil secondary flower phenotype (Venglat and Sawhney, 1996; Han et al., 2014). This secondary flower phenotype can be rescued by mutations of cytokinin receptors. Further molecular dissection showed that AP1 suppresses the cytokinin biosynthetic gene *LOG1* and activates the cytokinin degradation gene *CKX3*, through direct binding to the target gene promoters, thus reducing cytokinins levels in the outer whorls of developing flowers. Restoring the expression levels of either *LOG1* or *CKX3* can partially rescue the *ap1* secondary flower phenotype. In addition to affecting cytokinin homeostasis, AP1 also directly downregulates a group of flowering time-related MADS-box genes, including *SHORT VEGETATIVE PHASE* (*SVP*), *AGAMOUS-LIKE 24* (*AGL24*), and *SUPPRESSOR OF OVEREXPRESSION OF CO1* (*SOC1*), to suppress secondary FM formation. Similar to *IPT8* overexpression, overexpression of *SVP*, *AGL24,* or *SOC1* leads to sepal axil secondary FM formation (Liu et al., 2007). There appears to be crosstalk between cytokinin signaling and these flowering time-related MADS-box genes in the regulation of sepal axil secondary FM formation.

Taken together, the results described above show that cytokinins promote inflorescence complexity in different ways, by promoting meristem activity of IMs and BMs in inflorescences that branch iteratively, and by promoting indeterminate lateral meristem formation in inflorescences that branch laterally. Manipulating cytokinin levels directly or indirectly in crops is expected to change inflorescence complexity to increase yields (Kempin et al., 1995; Ashikari et al., 2005; Kurakawa et al., 2007; Zhang et al., 2012; Li et al., 2013).

## Conflict of Interest Statement

The authors declare that the research was conducted in the absence of any commercial or financial relationships that could be construed as a potential conflict of interest.
